# John Dacre

**Published:** 1945

**Authors:** 


### Obituary

### JOHN DACRE, M.R.C.S., L.R.C.P.

ON 30th December, 1944, the death occurred of Dr. John Dacre, who retired some years ago after being for many years a well-known practitioner in Clifton.

Dr. Dacre qualified in 1882, and in 1883 came to Bristol from Leeds Infirmary, where he had held the post of Assistant House Surgeon. In November of the same year he was elected Assistant Resident Medical Officer and Pathologist at Bristol Royal Infirmary. He later held the posts of Junior Resident Officer, House Physician, House Surgeon and Senior Resident Medical Officer.

For some years Dr. Dacre practised in Victoria Square, Clifton, and he became President of Bristol Medico-Chirurgical Society. During the 1914–18 War he served as Captain in the R.A.M.C. (T.) He had been a Consultant to the Children's Hospital.

He was formerly prominently associated with Bristol Medical Dramatic Club and also Clifton Operatic Society. He had a fine bass voice.

He leaves two sons.

